# Distribution of Glycated Haemoglobin According to Early-Life and Contemporary Characteristics in Adolescents and Adults without Diabetes: The 1982 and 1993 Pelotas Birth Cohorts

**DOI:** 10.1371/journal.pone.0162614

**Published:** 2016-09-14

**Authors:** Romina Buffarini, María Clara Restrepo-Méndez, Vera M. Silveira, Jaime J. Miranda, Helen D. Gonçalves, Isabel O. Oliveira, Bernardo L. Horta, Denise P. Gigante, Ana Maria Menezes, Maria Cecília F. Assunção

**Affiliations:** 1 Post-graduate Program in Epidemiology, Federal University of Pelotas, Marechal Deodoro 1160, 3rd floor, 96020–220, Pelotas, Brazil; 2 International Center for Equity in Health, Federal University of Pelotas, Rua Marechal Deodoro, 1160 3rd floor, 96020–220, Pelotas, Brazil; 3 Clinical Medical Department, Faculty of Medicine, Federal University of Pelotas, Rua Félix da Cunha 614, Centro, 96010–000, Pelotas, Brazil; 4 CRONICAS Center of Excellence in Chronic Diseases, Universidad Peruana Cayetano Heredia, Department of Medicine, School of Medicine, Lima, Peru; 5 Department of Physiology and Pharmacology, Federal University of Pelotas, Campus Universitário s/n, Capão do Leão, 96010–900, Pelotas, Brazil; 6 Department of Nutrition, School of Nutrition, Federal University of Pelotas, Campus Anglo, Gomes Carneiro 1, 96010–900, Pelotas, Brazil; Medical University Innsbruck, AUSTRIA

## Abstract

**Aim:**

Glycated haemoglobin (HbA_1c_), a marker of glucose control in individuals with diabetes mellitus, is also related with the incidence of cardiometabolic risk in populations free of disease. The aim of this study was to describe the distribution of HbA_1c_ levels according to early-life and contemporary factors in adolescents and adults without diabetes mellitus.

**Methods:**

HbA_1c_ was measured in adults aged 30 years and adolescents aged 18 years who are participants in the 1982 and 1993 Pelotas Birth Cohorts, respectively. Bivariate and multivariate analyses were performed to describe the HbA_1c_ mean values according to early-life and contemporary characteristics collected prospectively since birth.

**Results:**

The distribution of the HbA_1c_ was approximately normal in both cohorts, with a mean (SD) 5.10% (0.43) in the 1982 cohort, and 4.89% (0.50) in the 1993 cohort. HbA_1c_ mean levels were significantly higher in individuals self-reported as black/brown skin color compared to those self-reported as white in both cohorts. Parental history of diabetes was associated with higher HbA_1c_ mean in adults, while stunting at one year old presented an inverse relation with the outcome in adolescents. No other early and contemporary factors were associated with HbA_1c_ levels in adults or adolescents.

**Conclusions:**

We found a consistent relationship between HbA_1c_ and skin color in both cohorts. Further research is needed to understand the role of genomic ancestry on levels of HbA_1c_ concentrations which may inform policies and preventive actions for diabetes mellitus and cardiometabolic risk.

## Introduction

Glycated haemoglobin (HbA_1c_) indicates the average blood glucose level during the previous eight to twelve weeks and is positively related to the concentration of glucose in blood [1]. HbA_1c_ is recognized as the best index for long-term glucose control in diabetic patients [1]. Also, it is regarded as a useful screening tool for detecting diabetes in general population [2, 3]. In epidemiological studies, HbA_1c_ has been found to be associated with atherosclerosis [4, 5], incidence of diabetes [6], cardiovascular disease (CVD) [7–9], and all-cause mortality in adult population without diabetes [10–13].

The HbA_1c_ measurement offers some advantages compared with other glycemic indicators. It can be performed at any time of the day, irrespective of fasting or feeding, and it is relatively cheap [2, 14]. Therefore, HbA_1c_ is used to examine the disease risk associated to hyperglycaemia in healthy populations [15].

Birth weight and nutritional status in the two first years of life have been associated with different diseases as coronary heart disease, impaired glucose tolerance and diabetes [16–18]. Alcohol and tobacco use, physical inactivity, obesity, high waist circumference are well known risk factors for chronic diseases as well ([19, 20]. With the increase in metabolic conditions and the use of in HbA_1c_ as glycaemic indicator, a better understanding of the relation between HbA_1c_ concentrations and demographic, socioeconomic, behavioral and anthropometric characteristics is needed. This knowledge may help to identify groups of the population with increased risk of cardiometabolic diseases. Although the levels of HbA_1c_ have been described for adults and adolescents without diabetes [21, 22], the literature examining these associations in healthy populations is still scant.

In this study we aimed to describe the distribution of HbA_1c_ levels according to known early-life and contemporary cardiometabolic risk factors in adolescents and adults without diabetes who are participants of two population-based birth cohorts.

## Materials and Methods

### Study design and population

Pelotas is a middle-sized city located in the extreme south of Brazil. The estimated total population for 2014 was approximately 340,000 inhabitants. In 1982 and 1993, two birth cohort studies began, in which all the residents in the urban area of Pelotas municipality were eligible. Based on the high percentage of hospital deliveries in the city (always above 98%), all the city’s hospitals were daily visited by trained fieldwork team members. The mothers were interviewed soon after delivery using a structured questionnaire. Non-hospital deliveries were also included in the cohorts, since mothers normally sought a maternity ward after delivery, and were thus recruited to the study at this stage. The non-response rate at recruitment in both cohorts was below 1% [23]. Since then, the cohort members have been interviewed numerous times. On each occasion pre-tested standardized questionnaires were used and specially trained interviewers and field workers examined the subjects. Further detailed description of the methodology has been published previously [24–27].

Assessment of the outcome (HbA_1c_) relies on the last visit of each cohort, the 30 years follow-up for the 1982 Cohort and the 18 years follow-up for the 1993 Cohort, which were carried out in 2012–13 and 2011–12 respectively. In the 30 years wave, 4321 members were located, of whom 3701 were interviewed. Those who completed the interviews, added to those known to have died (n = 325), represented 68% of the original cohort. In the 18 years visit, 87% (n = 4563) of the original cohort was located, and 4106 of these were examined. Including those members known to have died (n = 164), the follow-up rate was 81,3%. These follow-ups were carried out at the university research clinic and included interview, physical examination, assessment of physical activity and collection of biological samples [28, 29].

Study protocols were approved by the Medical Research Ethics Committee of the Federal University of Pelotas. Verbal informed consent for participation in the study was provided by mothers until 1986 in the 1982 Cohort and in perinatal phase 1993 Cohort. Written full informed consent was obtained from parents (if the participant was under 18 years old) or by the participants themselves at the subsequent visits. A written consent was not obtained in the earlier phases of the 1982 and 1993 Pelotas cohort studies as in Brazil the written inform consent was established only in 1996 by the National Health Council. Nonetheless, before 1997, the research projects were built considering a previous national resolution (Brazilian Resolution No.01 of June 13th, 1988) and followed the main regulation of national and international codes.

### Outcome measurement

Blood samples were drawn by venipuncture and collected in ethylenediaminetetraacetic acid (EDTA) collection tubes in the day of the visit. Exclusion criteria for doing the blood drawing included pregnancy or probable pregnancy. Whole blood spot samples were prepared by pipetting 50 μL on to the filter paper card (Protein Saver ^TM^903® card, Whatman) and kept at room temperature for drying. After drying, the filter cards were kept in sealed foil bags including desiccant pouch and stored at -80°C.

HbA_1c_ was measured on whole blood samples by ion-exchange high-performance liquid chromatography (HPLC) method. Dried whole blood spots were prepared by cutting 3mm punches from each filter paper card sample. The elution of the sample was performed according to the protocol used in the VARIANT^TM^ II testing system (Bio-Rad Laboratories Inc, Hercules, CA). This analyzer was standardized by the Diabetes Control and Complications Trial reference method.The intraassay and interassay coefficients of variation were 0.9% and 3.4–5.1%, respectively. Results are shown in percentage of HbA_1c_ of total hemoglobin.

We have information on HbA_1c_ for 3543 participants in the 1982 Cohort (59.3% of the original cohort, and 96.7% of the followed-up in the 30 years visit); and 3831 in the 1993 Cohort (75% of the original cohort, and 93.3% of the followed-up in the 18 years visit). As we attempted to describe the distribution de HbA1c in population without diabetes, subjects on antidiabetic medication (oral hypoglycemic agents or insulin) and those who had values of Hba1c > = 6.5% (n = 36 and 26 in 1982 and 1993 cohorts, respectively) were excluded, which comprises the final sample included in our analyses.

### Independent variables

#### Early-life variables

Self reported skin color was collected based on the categories proposed by the Brazilian Institute of Geography and Statistics: white, black, brown, yellow and indigenous; and then grouped as white or black/brown. The participants who described themselves as “yellow” or “indigenous” were only 3% of the interviewed subjects and were removed from the analyses.

Birth weight, collected at perinatal follow-up, was measured by hospital staff using pediatric scales whose accuracy were periodically checked by the research team. Low birth weight (LBW) was defined as birth weight <2500 g.

Nutritional status indicators at one year old were based on data of subsamples of both cohorts. The 1982 cohort subsample included all infants who were born from January to April 1982 (n = 1916). In the 1993 cohort, all low birth weight (<2500 g) children plus 20% systematic sample of all other newborns were included (n = 1460). Supine length measurement was taken using AHRTAG portable infantometers with 1 mm precision (AHRTAG, London, UK), custom built for these studies. Weight was evaluated using Salter CMS mechanical scales with 25 kg maximum and 100 g precision (Salter, Tonbridge, United Kingdom). In both cohorts, scales were calibrated on a weekly basis using standard weights. Length-for-age, weight-for-length and body mass index-for-age *z-*scores were calculated according to the growth curves published by the World Health Organization in 2006.Children with z-scores of length-for-age and weight-for-length below −2 were classified as stunting and wasting, respectively. Overweight was defined as body mass index-for-age above +2 standards deviations.

#### Contemporary variables

The contemporary factors were assessed at the last waves of each cohort (30 and 18 years), with exception of parental history of diabetes on the 1993 cohort that was evaluated at the 11-year visit. Parental history of diabetes was assessed in both cohorts asking if one or both parents had diabetes. Monthly family income in Brazilian currency (categorized in tertiles) was used as a measure of socioeconomic position (SEP).

Behavioral characteristics included smoking status, alcohol consumption and physical inactivity. The regular alcohol consumption was measured in categories of number of daily drinks, with slight differences between both cohorts. While in the 1982 cohort the categories were 0–1, 2–3, 4–5, 6–7, 8 or more alcohol servings per day; in the 1993 cohort the responses were 0, 1–2, 3–4, 5–6, 7–8, and 9 or more. Physical activity was assessed using a validated questionnaire which includes questions related to mode of commuting to school and work, and activities practiced during leisure time. The total physical activity score was generated by the sum of minutes per week spent on leisure-time and commuting activities. Adolescents with physical activity practice below 300 minutes per week were considered inactive. For adults, the cut-off point to define physical inactivity was 150 minutes per week [30].

Current weight was measured to the nearest 0.1 kg on electronic scale TANITA (model BC‐418 MA; Tanita, Tokyo, Japan). Standing height was assessed to the nearest 0.1 cm using a wall‐mounted stadiometer (SECA 240; Seca, Birmingham, United Kingdom).Body mass index (BMI) was calculated by dividing weight by height squared (kg/m^2^) and cutoffs were defined separately for adults (20 y or more) [30],and for adolescents (10–19 y) [31, 32]. Due to small proportion of observations in the underweight category (less than 2% in each cohort), it was combined with the normal weight group. Waist circumference, categorized in tertiles, was evaluated with individuals in standing position using a flexible 160 cm (precision: 1 mm) fiberglass measuring tape. The measurement was taken at the narrowest point of the torso. Participants were barefoot and wearing light clothing.

### Statistical analyses

The distribution of HbA_1c_ in the sample studies was described using mean, standard deviation (SD) and histogram, and verified by values of skewness and kurtosis. As HbA_1c_ was sufficiently normally distributed in both samples any transformation was unnecessary.

In the bivariate analyses, we described the mean and standard error (SE) of HbA_1c_ according to categories of the independent variables. T-test or analyses of variance (ANOVA) were used to test for the significance of the mean differences in HbA_1c_ among categories. When appropriate, test for linear trends were performed. To assess the independent association of the outcome and each of the independent variables, multiple linear regression was performed. All independent variables were included in a fully-adjusted model regardless of their level of statistical significance in the bivariate analysis of the association with the outcome measure. The same confounding structure was used for adjustment to facilitate the comparison between cohorts, thus variables that show association for either both cohorts or for one of them were included in the multivariate models. Interaction between HbA_1c_ and sex was tested. As there was no strong evidence of heterogeneity of HbA_1c_ concentrations between boys and girls, the results are shown for both sexes together.

For the main analyses presented here, we only included individuals who had complete data on any variables included in the fully- adjusted models (restricted sample). To assess possible effects of missing data from loss to follow-up, unadjusted analyses were carried out comparing both the restricted sample used in the main analyses with the maximal sample available for each independent variable. Analysis using data of the 12-month follow-up of the 1993 were weighted to correct for the oversampling of low birth weight. All the analyses were performed using the software Stata version 12.1 (StataCorp, College Station, TX, USA).

## Results

Table 1 shows the description of the study samples. About half of the members were girls, and approximately two thirds were white. About 8% of the participants had low birthweight. At one year old, 8% of the 1982, and 13% of the 1993 cohort had chronic malnutrition; while both cohorts presented approximately 2% of wasting and 10% of overweight. The prevalence of parental history of diabetes was about one third in the 1982 cohort and 8% in the 1993 cohort. Concerning smoking, about 24% and 11% of the adults and adolescents were smokers, respectively. In both cohorts, about 7% of the participants drunk nine or more alcohol servings per day, and about 40% were physically inactive. The prevalence of overweight and obesity was observed in more than a half of the 1982 cohort members, and less than one third in the 1993 cohort.

**Table 1:** Characteristics of participants with information on normal values of HbA_1c_*. 1982 and 1993 Pelotas Birth Cohorts.

Independent variables	1982 cohort		1993 cohort	
	N	%	N	%
**Early-life characteristics**				
Sex				
Girls	1778	50.7	1892	49.7
Boys	1729	49.3	1913	50.3
Skin Color				
White	2664	78.5	2340	66.4
Black and brown	731	21.5	1182	33.6
Low birth weight				
No	3256	92.9	3459	90.9
Yes	250	7.1	346	9.1
Stunting (<-2 height/age)**				
No	863	92.2	992	87.2
Yes	73	7.8	135	12.8
Wasting (<-2 weight/height)**				
No	923	98.6	1407	99.0
Yes	13	1.4	10	1.0
Overweight (>2 bmi/age)**				
No	874	93.4	961	90.9
Yes	62	6.6	96	9.1
**Contemporary characteristics**				
Parental history of diabetes***				
No	1960	67.8	3317	91.7
Yes	933	32.3	301	8.3
Family income (tertiles				
1 (poorer)	1133	34.1	1282	33.6
2	1093	33.0	1243	32.9
3 (richest)	1086	32.9	1280	33.5
Smoking				
Non-smokers	2027	58.5	2954	77.7
Ex-smokers	614	17.7	312	8.2
Smokers	824	23.8	536	14.1
Alcohol intake (servings per day)				
0 to 2	794	30.5	1832	65.0
3 to 8	1611	61.8	796	28.2
9 or more	200	7.7	192	6.8
Physical inactivity				
No	1992	58.0	2319	61.1
Yes	1444	42.0	1477	38.9
Nutritional status				
Underweight and normal	1478	42.5	2752	72.8
Overweight	1204	34.6	651	17.2
Obesity	797	22.9	375	9.9
Waist circumference (tertiles)				
1 (lowest)	1165	33.4	1269	33.4
2	1165	33.3	1266	33.4
3 (highest)	1160	33.3	1259	33.2

* Less than 6.5% or taking antidiabetic medication

**One year old follow-up

*** 1993 cohort: eleven year old follow-up

The distribution of independent variables in the total number of cohort members who participated in the last visits is shown in Table A in S1 File. The prevalence of low birth weight (7%) and men (49.3%) in the 1982 cohort are slightly underrepresented in the study sample compared with the total number of participants at the last visit (9% and 51.4% for low birth weight and men, respectively). No other differences were found between these samples characteristics in the cohorts.

The mean age was 30.2 years for the 1982 cohort participants and 18.5 years for the 1993 cohort members. The distributions of HbA_1c_ levels were approximately normal in both cohorts with a higher mean among individuals from the 1982 cohort than among those from the 1993 cohort (5.10% vs. 4.89%; p<0.001). The dispersion of HbA_1c_ values, as reflected by the standard deviations, was larger in the 1993 cohort compared with the 1982 cohort (0.43% vs. 0.50%).

### 1982 cohort

The association between HbA_1c_ levels and early and contemporary factors for adults members of the 1982 cohort are shown in Table 2. No mean differences were found for any of the indicators of nutritional status at aged 1-year-old. On the other hand, higher HbA_1c_ mean levels were found among black/brown compared with white (5.1 9 vs. 5.09 p = 0.023), and among those whose mother and/or father had diabetes (5.19 vs. 5.08 p = 0.006). Monthly familiar income and behavioral characteristics such as alcohol intake, smoking status and physical inactivity were not associated with HbA_1c_ levels. HbA_1c_ means increased progressively from the lowest to the highest categories of BMI (p = 0.036), being those classified as obese who presented the highest level of HbA_1c_.

**Table 2:** Unadjusted and adjusted mean and SE for HbA_1c_ according to early-life, demographic, socioeconomic and behavioral factors, parent history of diabetes, nutritional status and waist circumference among adults in the 1982 Pelotas Birth Cohort.

Independent variables	N	1982 Cohort			
		Unadjusted		Adjusted	
		Mean (SE)	p-value	Mean (SE)	p-value
**Early-life characteristics**					
Sex			0.808 ^a^		0.442 ^c^
Girls	291	5.10 (0.02)		5.10 (0.03)	
Boys	270	5.11 (0.03)		5.13 (0.03)	
Skin Color			0.023 ^a^		0.044 ^c^
White	455	5.09 (0.02)		5.10 (0.02)	
Black and brown	106	5.19 (0.04)		5.18 (0.03)	
Low birth weight			0.308 ^a^		0.249 ^c^
No	533	5.11 (0.02)		5.12 (0.02)	
Yes	28	5.05 (0.10)		5.02 (0.08)	
Stunting (<-2 height/age)*			0.294 ^a^		0.347 ^c^
No	527	5.12 (0.02)		5.12 (0.02)	
Yes	34	5.04 (0.07)		5.05 (0.08)	
Wasting (<-2 weight/height)*			0.139 ^a^		0.075 ^c^
No	554	5.11 (0.02)		5.11 (0.02)	
Yes	7	5.34 (0.11)		5.40 (0.16)	
Overweight (>2 bmi/age)*			0.927 ^a^		0.668 ^c^
No	525	5.11 (0.02)		5.12 (0.02)	
Yes	56	5.12 (0.07)		5.08 (0.07)	
**Contemporary characteristics**					
Parental history of diabetes			0.006^a^		0.017 ^c^
No	399	5.08 (0.02)		5.09 (0.02)	
Yes	162	5.19 (0.03)		5.18 (0.03)	
Family income (tertiles)			0.972^a^		0.548 ^c^
1 (poorer)	166	5.12 (0.03)		5.10 (0.03)	
2	172	5.12 (0.03)		5.11 (0.03)	
3 (richest)	223	5.11 (0.02)		5.12 (0.03)	
Smoking			0.483 ^a^		0.410 ^b^
Non-smokers	341	5.11 (0.02)		5.10 (0.02)	
Ex-smokers	100	5.09 (0.04)		5.11 (0.04)	
Smokers	120	5.15 (0.04)		5.15 (0.04)	
Alcohol intake (servings per day)			0.956 ^a^		0.949 ^c^
0 to 1	181	5.10 (0.03)		5.12 (0.02)	
2 to 7	339	5.11 (0.02)		5.11 (0.03)	
8 or more	41	5.12 (0.06)		5.11 (0.07)	
Physical inactivity			0.239 ^a^		0.220 ^c^
No	328	5.10 (0.02)		5.10 (0.02)	
Yes	233	5.13 (0.03)		5.14 (0.03)	
Nutritional status			0.036^b^		0.136 ^b^
Underweight and normal	228	5.08 (0.03)		5.05 (0.02)	
Overweight	194	5.12 (0.03)		5.15 (0.02)	
Obesity	139	5.17 (0.03)		5.17 (0.02)	
Waist circumference (tertiles)			0.123		0.476^b^
1 (lowest)	180	5.11 (0.03)		5.07 (0.05)	
2	190	5.07 (0.03)		5.07 (0.03)	
3 (highest)	191	5.16 (0.03)		5.11 (0.04)	

HbA_1c_ shown as percentage of total haemoglobin

SE: standard error

Adjusted for all the independent variables

*Subsample at one year-old follow-up (1983)

^a^ T test or ANOVA

^b^ Linear trend

^c^ Wald test

The unadjusted results using the maximum sample available (Table B in S1 File) were compared with those presented here in the main analyses (only included individuals with complete data on outcome and all independent variables). In general, associations in the maximal sample were similar to those observed in the restricted sample, except for significant differences between HbA_1c_ and the independent variables wasting at 1-year-old and current waist circumference.

After adjustment for all early and contemporary characteristics, only skin color and parental history of diabetes remained associated with HbA1c mean levels (p = 0.044 and p = 0.017, respectively).

### 1993 cohort

Table 3 shows the association between HbA_1c_ levels and early and contemporary factors among adolescents members of the 1993 cohort. HbA_1c_ levels were not associated with sex, low birth weight, wasting and overweight. However, those classified as stunted at 1-year-old, showed lower mean levels of HbA_1c_ in adolescence than those classified as not stunted (4.75 vs. 4.89 p = 0.029) Mean HbA_1c_ levels were higher among black/brown skin color compared with white skin color (4.95 vs. 4.86 p = 0.016). There was no difference in HbA_1c_ mean levels according to parental history of diabetes, tertiles of family income, alcohol drinking, smoking, physical inactivity and BMI categories. Mean HbA_1c_ levels were linearly higher with increasing tertiles of waist circumference (p = 0.008). In the multivariate model, only skin color and stunting remained associated with HbA_1c_ concentrations (p = 0.023 and p = 0.013, respectively).

**Table 3:** Unadjusted and adjusted mean and SE for HbA_1c_ according to early-life, demographic, socioeconomic and behavioral factors, parent history of diabetes, nutritional status and waist circumference among adolescents in the 1993 Pelotas Birth Cohort.

Independent variables	N	1993 Cohort			
		Unadjusted		Adjusted	
		Mean (SE)	p-value	Mean (SE)	p-value
**Early-life characteristics**					
Sex			0.085^a^		0.054^c^
Girls	352	4.86 (0.03)		4.83 (0.01)	
Boys	355	4.92 (0.03)		4.93 (0.01)	
Skin Color			0.016^a^		0.023^c^
White	471	4.86 (0.02)		4.85 (0.01)	
Black and brown	236	4.95 (0.03)		4.95 (0.02)	
Low birth weight			0.289 ^a^		0.082 ^c^
No	493	4.88 (0.02)		4.87 (0.02)	
Yes	214	4.92 (0.04)		4.95 (0.04)	
Stunting (<-2 height/age)*			0.029 ^a^		0.013 ^c^
No	615	4.89 (0.02)		4.89 (0.02)	
Yes	92	4.75 (0.06)		4.71 (0.07)	
Wasting (<-2 weight/height)*			0.194^a^		0.296 ^c^
No	704	4.87 (0.02)		4.88 (0.02)	
Yes	3	5.21 (0.26)		5.14 (0.25)	
Overweight (>2 bmi/age)*			0.282 ^a^		0.127 ^c^
No	640	4.89 (0.02)		4.89 (0.02)	
Yes	67	4.82 (0.06)		4.80 (0.06)	
**Contemporary characteristics**					
Parental history of diabetes			0.837^a^		0.776^c^
No	649	4.89 (0.02)		4.89 (0.01)	
Yes	58	4.91 (0.06)		4.93 (0.03)	
Family income (tertiles)			0.483^b^		0.522^c^
1 (poorer)	218	4.92 (0.03)		4.90 (0.02)	
2	240	4.86 (0.03)		4.87 (0.02)	
3 (richest)	249	4.91 (0.03)		4.89 (0.02)	
Smoking			0.074^b^		0.170^c^
Non-smokers	516	4.89 (0.02)		4.89 (0.01)	
Ex-smokers	67	4.78 (0.06)		4.78 (0.03)	
Smokers	124	4.84 (0.04)		4.88 (0.02)	
Alcohol intake (servings per day)			0.420^a^		0.287 ^b^
0 to 2	445	4.91 (0.02)		4.91 (0.03)	
3 to 8	214	4.85 (0.03)		4.84 (0.04)	
9 or more	48	4.88 (0.07)		4.82 (0.07)	
Physical inactivity			0.506 ^a^		0.388^c^
No	443	4.90 (0.02)		4.87 (0.01)	
Yes	264	4.87 (0.03)		4.88 (0.01)	
Nutritional status			0.316^a^		0.586^b^
Underweight and normal	520	4.87 (0.02)		4.90 (0.01)	
Overweight	114	4.94 (0.05)		4.86 (0.03)	
Obesity	73	4.93 (0.06)		4.80 (0.03)	
Waist circumference (tertiles)			0.008^b^		0.093^b^
1 (lowest)	252	4.84 (0.03)		4.83 (0.02)	
2	231	4.86 (0.03)		4.84 (0.02)	
3 (highest)	224	4.97 (0.03)		4.98 (0.02)	

HbA_1c_ shown as percentage of total haemoglobin

SE: standard error

Adjusted for all the independent variables

*Subsample at one year-old follow-up (1994)

^a^ T testor ANOVA

^b^ Linear trend

^c^ Wald test

In the crude analyses using the maximal sample available for each independent variable (Table C in S1 File), significant differences were found between HbA_1c_ and sex, alcohol drinking and physical activity; while stunting was not related to the outcome.

## Discussion

We have described the distributions and mean values of HbA_1c_ according to demographic, socioeconomic, behavioral and anthropometric characteristics in two nondiabetic population-based cohorts. Our results showed higher HbA_1c_ means black/brown individuals in both cohorts. Besides, we observed positive association between HbA_1c_ mean levels and having parental history of diabetes in adult members of the 1982 cohort; and being not stunting at 1 year old in adolescents members of the 1993 cohort.

This study found higher HbA_1c_ levels in the 30 years old cohort members compared with the 18 years old cohort members. The increase of HbA_1c_ with ageing has been observed in other studies [33] [21]. Gulliford et al. observed a 0.12% increased in glycated haemoglobin every ten years of age in a population that included individuals aged 16 years or more [33]. Studies that assessed adult and elderly population showed a positive association HbA_1c_ and age that remained significant after adjustment for BMI [21, 34]. However, the relationship between HbA_1c_ and ageing in younger populations is controversial. [22, 35]. A study examined children and adolescents from four to seventeen years and demonstrated a linear association between HbA_1c_ and age only in females and African-American males [35]; while other reported no linear trend in a sample with participants aged five to 24 years old [22]. A previous study carried out with a subsample of males members of the 1982 cohort when participants were 18 year old reported a mean HbA_1c_ of 5.22% [36], which it is higher than the one we found in this study in adolescents members of the 1993 cohort, and even higher than the current HbA_1c_ concentrations in the male participants of the 1982 cohort. This difference may be explained by variation among different assay methods.

The positive association between black individuals and HbA_1c_ among diabetics or those with glucose impaired tolerance is well established in the literature showing higher HbA_1c_ concentrations in African Americans relative to non-Hispanic Whites [37–39]. Our results support the finding that skin color is related with HbA_1c_ values even among young nondiabetic populations [22, 35], which it is in line with observational studies that examined adults without diabetes and found higher HbA_1c_ means in black relative to white individuals [40, 41]. The higher HbA_1c_ mean levels in blacks versus whites persist even after adjustment for SEP has been reported in various studies as well. The skin color differences may reflect an adverse profile of parameters related to glucose homeostasis such as insulin sensitivity and secretion [42] or higher prevalence of type 2 diabetes mellitus among minorities groups [43]. Reasons for skin color differences in HbA_1c_ concentrations remain unclear [44]. An important issue to be addressed in extensive studies is whether the observed skin color differences in levels of hemoglobin glycation reflect a greater risk for cardiometabolic diseases in black people compared with whites.

Low birthweight is known to be related with an adverse glucose and insulin profile in adult life [45], although, the relationship is less clear regarding HbA_1c_. In line with our findings, most of studies did not find any association between birthweight and HbA_1c_ in children [46], adolescents [36] and adults [47]; while a recent study carried out in English children found a 0.04% increase in HbA_1c_ for every 100 g of lower birthweight [48]. The mentioned association appeared after adjustment for current height and became stronger with further adjustment for body fatness, indicating that the association depends on childhood size more than birthweight per se. On other hand, wasting and stunting at first year of life were associated with HbA_1c_ mean levels. In the 1982 cohort we observed that those adults who were classified as wasted at first year of life, presented higher mean HbA_1c_ levels. However, this association disappeared in the adjusted model which may be explained by the small prevalence of wasting (1.4%). In addition, we observed an inverse association between stunting at first year of life and HbA_1c_ mean levels in adolescent members of the 1993 cohort, which it is an unexpected finding considering previous research on the developmental origins of cardiometabolic conditions. Studies suggested that individuals who are small in the first years of life and subsequently put on weight rapidly present the greatest levels of risk for several metabolic conditions as coronary heart disease [49, 50], impaired glucose tolerance [51] and blood pressure [52]. We cannot ruled out the possibility of residual confounding as this result was not consistent to that observed for adults in the 1982 cohort.

Previous studies showed positive association with family history of diabetes, a well known risk factor for development of diabetes, in adults [21, 40] as well in adolescents [22]. This finding, which it is consistent with what we found in the 1982 cohort, indicate that there are possibly genetic factors involved in this relationship. However, non-significant results were found in the 1993 cohort. The discrepancy in findings for parental history of diabetes between both cohorts may reflect differences in the parent’s age of the cohort members. In the 1993 cohort the variable was assessed when the adolescents were eleven years old, thus, the parents the cohort members were quite younger than parents from the 1982 cohort members (in which the question was asked at the age of 30). This may be related to the low diabetes prevalence observed among parents from the 1993 cohort and, as a result, to the absent of association with HbA_1c_ levels in the adolescents.

No mean differences were observed between family income and HbA_1c_ in the current analysis. Even with diverse socioeconomic indicators, other reports have also demonstrated weak relationship between SEP and HbA_1c_ in adolescents [22, 35]. The lack of association between familiar income and HbA_1c_ may be due to the fact that socioeconomic level could have a delayed effect on physical health in relation to chronic conditions. Therefore, their effects are not evident at early ages (e.g. younger than 35 years). It has been suggested that the effect of socioeconomic inequalities on biological outcomes emerge later in life, partly linked to different indirect pathways [53]. These may also explain the lack of association between behavioral factors and HbA_1c_. Further studies of these cohorts later in life will be needed to test this hypothesis.

Our findings are consistent with data from the NHANES III regarding the association between HbA_1c_ levels and BMI which is not significant after controlling for covariates such as sex, race, maternal BMI and socioeconomic status [35]. On the other hand, BMI was shown to be an independent correlate of HbA_1c_ concentrations in Japanese middle-aged and elderly population [54]. Furthermore, waist circumference have also been positively related with HbA_1c_ in the Japanese study [54] and in the Bogalusa cohort in the United States [40]. We observed positive trends of HbA_1c_ means according to increasing tertiles of waist circumference, however this association disappeared after adjustment for covariates. Again, this discrepancy of our results in relation to previous studies may be explained by age. We examined a younger population than Nguyen et al. which examined adults aged 32 to 40 years, and Yoshida et al. which assessed individuals aged 50 to 74 year. Given the importance of the waist circumference as a predictor of CVD and type 2 diabetes [55], the lack of statistical significance is not a reason to not give attention to the mean differences we have found.

Given that the present study had *a priori* samples sizes, the minimum detectable differences were calculated with a 5% of alpha error and 80% of power. Based on these parameters, the study would have sufficient power to detect differences of HbA_1c_ levels between 0.08 and 0.45 for early-life factors, and 0.05 and 0.12 for contemporary variables. As a limitation, it is important to mention the fact that most of the associations may have been underpowered to detect the minimum mean differences of HbA_1c._ Furthermore, when we examined the unadjusted differences between the outcome and categories of the independent variables in both the restricted and maximal samples, we found more significant associations using the maximal samples (i.e. not restricting results to only those with complete data in the outcome and all covariates) in the unadjusted analyses. This may add evidence to the hypothesis of low power due to smaller sample size. However, we cannot rule out the possibility of bias due to missing data.

The strengths of our report include the two large population-based cohorts in middle-income setting and the assessment several independent variables, including early and contemporary life factors. It is important to highlight that the HbA_1c_ measurement was evaluated for nearly 60% of the 1982 cohort members and 75% of the 1993 cohort members, which are high percentages of follow-up.

To sum up, we showed representative data on HbA_1c_ distributions among individuals of 18 and 30 years old without diabetes mellitus belonging to two cohorts of southern Brazil. We found normal distributions of HbA_1c_ values and a consistent relationship between HbA_1c_ and self-assessed skin color in both cohorts. These findings suggest that more research is needed to understand the role of genomic ancestry on levels of HbA_1c_ concentrations.

## Supporting Information

S1 File Supporting information. (DOCX)
