# Supplementary material for: Distribution of Glycated Haemoglobin According to Early-Life and Contemporary Characteristics in Adolescents and Adults without Diabetes: The 1982 and 1993 Pelotas Birth Cohorts
Source: PLoS One. 2016 Sep 14;11(9):e0162614. doi: 10.1371/journal.pone.0162614 (PMC5023185; doi:10.1371/journal.pone.0162614)
Supplement: S1 File — (DOCX) [file pone.0162614.s001.docx]

**Supporting information**

**Table A.** Characteristics of participants who were evaluated at the 30 and 18 years old visits, 1982 and 1993 Pelotas Birth Cohorts, respectively.

| Independent variables | 1982 cohort | | 1993 cohort | |
| --- | --- | --- | --- | --- |
|  | N | % | N | % |
| **Early-life characteristics** | | | | |
| Sex |  |  |  |  |
| Girls | 2876 | 48.6 | 2642 | 50.3 |
| Boys | 3037 | 51.4 | 2606 | 49.7 |
| Skin Color |  |  |  |  |
| White | 2817 | 78.6 | 2769 | 66.5 |
| Black and brown | 768 | 21.4 | 1395 | 33.5 |
| Low birth weight |  |  |  |  |
| No | 5375 | 91.0 | 4739 | 90.3 |
| Yes | 534 | 9.0 | 510 | 9.7 |
| Stunting (<-2 height/age)* |  |  |  |  |
| No | 1333 | 91.5 | 1179 | 86.6 |
| Yes | 124 | 8.5 | 182 | 13.4 |
| Wasting (<-2 weight/height)* |  |  |  |  |
| No | 1427 | 97.9 | 1344 | 98.7 |
| Yes | 30 | 2.1 | 17 | 1.3 |
| Overweight (>2 bmi/age)* |  |  |  |  |
| No | 1356 | 93.1 | 1231 | 90.4 |
| Yes | 101 | 6.9 | 130 | 9.6 |
| **Contemporary characteristics** | | | | |
| Parental history of diabetes** |  |  |  |  |
| No | 2053 | 67.7 | 3987 | 91.8 |
| Yes | 980 | 32.3 | 354 | 8.2 |
| Family income (tertiles |  |  |  |  |
| 1 (poorer) | 1191 | 34.2 | 1404 | 34.2 |
| 2 | 1137 | 32.6 | 1336 | 32.5 |
| 3 (richest) | 1155 | 33.2 | 1366 | 33.3 |
| Smoking |  |  |  |  |
| Non-smokers | 2139 | 58.7 | 3179 | 77.5 |
| Ex-smokers | 647 | 17.7 | 354 | 8.6 |
| Smokers | 860 | 23.6 | 570 | 13.9 |
| Alcohol intake (servings per day) |  |  |  |  |
| 0 to 2 | 836 | 30.8 | 1954 | 64.8 |
| 3 to 8 | 1669 | 61.6 | 859 | 28.5 |
| 9 or more | 205 | 7.6 | 203 | 6.7 |
| Physical inactivity |  |  |  |  |
| No | 2091 | 58.0 | 2495 | 60.9 |
| Yes | 1515 | 42.0 | 1600 | 39.1 |
| Nutritional status |  |  |  |  |
| Underweight and normal | 1509 | 42.5 | 2881 | 72.7 |
| Overweight | 1228 | 34.6 | 680 | 17.2 |
| Obesity | 814 | 22.9 | 400 | 10.1 |
| Waist circumference (tertiles) |  |  |  |  |
| 1 (lowest) | 1189 | 33.3 | 1330 | 33.4 |
| 2 | 1192 | 33.4 | 1325 | 33.3 |
| 3 (highest) | 1186 | 33.3 | 1322 | 33.3 |

* One year old follow-up

** 1993 cohort: eleven year old follow-up

**Table B.** Unadjusted mean and SE for HbA_1c_ according to early-life, demographic, socioeconomic and behavioral factors, parent history of diabetes, nutritional status and waist circumference among adults in the 1982 Pelotas Birth Cohort in the maximal sample.

| Independent variables | N | Mean (SE) | p-value |
| --- | --- | --- | --- |
| **Early- life characteristics** |  |  |  |
| Sex |  |  | 0.058^a^ |
| Girls | 1778 | 5.09 (0.01) |  |
| Boys | 1729 | 5.11 (0.01) |  |
| Skin Color |  |  | 0.039 ^a^ |
| White | 2664 | 5.09 (0.01) |  |
| Black and brown | 731 | 5.13 (0.02) |  |
| Low birth weight |  |  | 0.585 |
| No | 3256 | 5.10 (0.01) |  |
| Yes | 250 | 5.11 (0.03) |  |
| Stunting (<-2 height/age)* |  |  | 0.570 |
| No | 863 | 5.12 (0.14) |  |
| Yes | 73 | 5.09 (0.05) |  |
| Wasting (<-2 weight/height)* |  |  | 0.012 |
| No | 923 | 5.11 (0.01) |  |
| Yes | 13 | 5.40 (0.07) |  |
| Overweight (>2 bmi/age)* |  |  | 0.495 |
| No | 874 | 5.12 (0.01) |  |
| Yes | 62 | 5.08 (0.06) |  |
| **Contemporary characteristics** | |  |  |
| Parental history of diabetes |  |  | 0.003 ^a^ |
| No | 1960 | 5.09 (0.01) |  |
| Yes | 933 | 5.14 (0.01) |  |
| Family income (tertiles) |  |  | 0.445 ^a^ |
| 1 (poorer) | 1133 | 5.08 (0.01) |  |
| 2 | 1093 | 5.10 (0.01) |  |
| 3 (richest) | 1086 | 5.10 (0.01) |  |
| Smoking |  |  | 0.687 ^a^ |
| Non-smokers | 2027 | 5.09 (0.01) |  |
| Ex-smokers | 614 | 5.11 (0.02) |  |
| Smokers | 824 | 5.10 (0.02) |  |
| Alcohol intake (servings per day) |  |  | 0.082 ^a^ |
| 0 to 1 | 1847 | 5.08 (0.02) |  |
| 2 to 7 | 558 | 5.11 (0.01) |  |
| 8 or more | 200 | 5.10 (0.03) |  |
| Physical inactivity |  |  | 0.498 ^a^ |
| No | 1992 | 5.09 (0.01) |  |
| Yes | 1444 | 5.10 (0.01) |  |
| Nutritional status |  |  | <0.001^b^ |
| Underweight and normal | 1478 | 5.07 (0.01) |  |
| Overweight | 1204 | 5.10 (0.01) |  |
| Obesity | 797 | 5.15 (0.02) |  |
| Waist circumference (tertiles) |  |  | <0.001^b^ |
| 1 (lowest) | 1165 | 5.06 (0.01) |  |
| 2 | 1162 | 5.10 (0.01) |  |
| 3 (highest) | 1160 | 5.14 (0.01) |  |

HbA_1c_ shown as percentage of total haemoglobin

SE: standard error

Adjusted for all the independent variables

*Subsample at one year-old follow-up (1983)

^a^ T test or ANOVA

^b^ Linear trend

^c^  Wald test

**Table C.** Unadjusted mean and SE for HbA_1c_ according to early-life, demographic, socioeconomic and behavioral factors, parent history of diabetes, nutritional status and waist circumference among adolescents in the 1993 Pelotas Birth Cohort in the maximal sample.

| Independent variables | N | Mean (SE) | p-value |
| --- | --- | --- | --- |
| **Early-life characteristics** |  |  |  |
| Sex |  |  | <0.001 ^a^ |
| Girls | 1892 | 4.83 (0.01) |  |
| Boys | 1913 | 4.95 (0.01) |  |
| Skin Color |  |  | 0.001 ^a^ |
| White | 2340 | 4.87 (0.01) |  |
| Black and brown | 1182 | 4.92 (0.02) |  |
| Low birth weight |  |  | 0.438 |
| No | 3462 | 4.89 (0.01) |  |
| Yes | 347 | 4.91 (0.03) |  |
| Stunting (<-2 height/age)* |  |  | 0.159 |
| No | 922 | 4.89 (0.02) |  |
| Yes | 135 | 4.80 (0.06) |  |
| Wasting (<-2 weight/height)* |  |  | 0.275 |
| No | 1047 | 4.88 (0.02) |  |
| Yes | 10 | 5.07 (0.18) |  |
| Overweight (>2 bmi/age)* |  |  | 0.387 |
| No | 961 | 4.88 (0.02) |  |
| Yes | 96 | 4.84 (0.05) |  |
| **Contemporary characteristics** |  |  |  |
| Parental history of diabetes |  |  | 0.155 ^a^ |
| No | 3317 | 4.89 (0.01) |  |
| Yes | 301 | 4.93 (0.03) |  |
| Family income (tertiles) |  |  | 0.577^b^ |
| 1 (poorer) | 1282 | 4.89 (0.01) |  |
| 2 | 1243 | 4.90 (0.01) |  |
| 3 (richest) | 1280 | 4.88 (0.01) |  |
| Smoking |  |  | 0.698^b^ |
| Non-smokers | 2954 | 4.89 (0.01) |  |
| Ex-smokers | 312 | 4.89 (0.03) |  |
| Smokers | 536 | 4.91 (0.02) |  |
| Alcohol intake (servings per day) |  |  | 0.024 ^a^ |
| 0 to 2 | 1832 | 4.90 (0.01) |  |
| 3 to 8 | 796 | 4.88 (0.02) |  |
| 9 or more | 192 | 4.99 (0.03) |  |
| Physical inactivity |  |  | 0.024 ^a^ |
| No | 2319 | 4.90 (0.01) |  |
| Yes | 1477 | 4.87 (0.01) |  |
| Nutritional status |  |  | 0.258 ^b^ |
| Underweight and normal | 2752 | 4.89 (0.01) |  |
| Overweight | 651 | 4.90 (0.02) |  |
| Obesity | 375 | 4.92 (0.03) |  |
| Waist circumference (tertiles) |  |  | <0.001^b^ |
| 1 (lowest) | 1269 | 4.85 (0.01) |  |
| 2 | 1266 | 4.90 (0.01) |  |
| 3 (highest) | 1260 | 4.92 (0.01) |  |

HbA_1c_ shown as percentage of total haemoglobin

SE: standard error

Adjusted for all the independent variables

*Subsample at one year-old follow-up (1994)

^a^ T testor ANOVA

^b^ Linear trend

^c^  Wald test
